# Tear Mediators in Corneal Ectatic Disorders

**DOI:** 10.1371/journal.pone.0153186

**Published:** 2016-04-13

**Authors:** Dorottya Pásztor, Bence Lajos Kolozsvári, Adrienne Csutak, András Berta, Ziad Hassan, Bernadett Ujhelyi, Péter Gogolák, Mariann Fodor

**Affiliations:** 1 Department of Ophthalmology, Faculty of Medicine, University of Debrecen, Debrecen, Hungary; 2 Orbident Refractive Surgery and Medical Center, Debrecen, Hungary; 3 Department of Immunology, Faculty of Medicine, University of Debrecen, Debrecen, Hungary; University of Oklahoma Health Sciences Center, UNITED STATES

## Abstract

**Purpose:**

To compare the concentrations of 11 tear mediators in order to reveal the biochemical difference between pellucid marginal degeneration (PMD) and keratoconus (KC).

**Methods:**

We have designed a cross-sectional study in which patients with corneal ectasia based on slit-lamp biomicroscopy and Pentacam HR (keratometry values (K1, K2, Kmax), astigmatism, minimal radius of curvature (Rmin), corneal thickness (Apex and Min), indices (surface variation, vertical asymmetry, keratoconus, central keratoconus, height asymmetry and decentration)) were enrolled. Eyes of keratoconic patients were similar to the PMD patients in age and severity (K2, Kmax and Rmin). Non-stimulated tear samples were collected from nine eyes of seven PMD patients, 55 eyes of 55 KC patients and 24 eyes of 24 healthy controls. The mediators’ (interleukin -6, -10, chemokine ligand 5, -8, -10, matrix metalloproteinase (MMP) -9, -13, tissue inhibitor of metalloproteinases (TIMP)-1, tissue plasminogen activator, plasminogen activator inhibitor, nerve growth factor) concentrations were measured using Cytometric Bead Array.

**Results:**

MMP-9 was the only mediator which presented relevant variances between the two patient groups (p = 0.005). The ratios of MMP-9 and TIMP-1 were 2.45, 0.40 and 0.23 in PMD, KC and the controls, respectively.

**Conclusion:**

As far as we are aware, this is the first study that aims to reveal the biochemical differences between PMD and KC. Further studies of biomarkers to investigate the precise role of these mediators need to be defined, and it is important to confirm the observed changes in a larger study to gain further insights into the molecular alterations in PMD.

## Introduction

Corneal ectatic disorders have considerable importance in public health [1]. They are characterized by progressive deformation of the corneal architecture—including corneal thinning at different locations—depending on the type of the ectasia. The rate of progression among the various ectatic entities is different and is associated with an increase in the spherical component of the refraction and irregular astigmatism with consequent deterioration of visual acuity, furthermore also results in the inducement of significant higher-order aberrations (HOAs), which contribute to a poorer retinal image quality, resulting in decreased visual acuity [2]. Pellucid marginal degeneration (PMD) is a very rare peripheral thinning disorder of the inferior (in atypical cases of the superior [3]) cornea, while keratoconus (KC) is the most common primary corneal ectatic disease that gives rise to a cone-shaped cornea [4–7]. It is not known whether PMD and KC are distinct diseases or whether they represent different clinical presentations of the same underlying disease process [4, 5, 8]. In early cases of PMD, the cornea may look relatively normal and, in severe cases, PMD (end-stage ectasias with large corneal scarring) may be difficult to differentiate from KC [8]. Pellucid marginal degeneration is often misdiagnosed and is often confused with inferior keratoconus [7–13]. PMD is usually discovered in the later decades of life (between the second and the fifth) compared to KC [5]. It is important to note that PMD is not associated with vascularisation and lipid or iron deposition [4, 5, 7]. The clinical significance of distinct PMD compared to KC is that the clinical continuum and treatment modalities—including the surgical interventions for these two entities—are different [14–16]. The adequate therapy depends on the stage of the ectasia [8]. In the early stages of both conditions, the management is most commonly non-surgical: KC is typically managed with rigid-gas permeable (RGP) contact lenses, whereas early cases of PMD are typically managed using spectacle lenses alone. In progressive cases, collagen cross-linking could be effective in both disorders [17]. Intracorneal ring segment implantation is widely used in KC, but may not be applicable in true PMD [13]. In advanced cases, both ectasias can be treated with penetrating or deep anterior lamellar keratoplasty, but the technical disposal is different (graft size, position) [18, 19]. Furthermore, crescentic wedge resection in PMD can also be a treatment [8, 19].

All histopathological studies of PMD and KC have noted the absence of inflammatory cells, and corneal ectatic disorders are generally believed to be non-inflammatory diseases with a multivariable origin [5, 8]. However, a recent review by Galvis and colleagues [20] has suggested that ectatic disorders are partly inflammatory conditions.

Biomarkers in the tear film have been studied in more depth in patients with KC but no studies have been reported for PMD. Studies have shown that the degradation of stromal collagen in KC is accompanied by the altered expression of cytokines, matrix metalloproteinases (MMPs; MMP-1, -3, -7,- 9, -13) with their inhibitors (tissue inhibitor of metalloproteinases-1 (TIMP-1)) and growth factors (epidermal growth factor (EGF), vascular endothelial growth factor (VEGF) as well as nerve growth factor (NGF)) [21–27]. In tears, altered levels of interferon (IFN)-γ, interleukin (IL)-4, IL-5, IL-6, IL-8 (chemokine (C-X-C motif) ligand (CXCL)8/IL-8), IL-10, IL-12, IL-13, chemokine (C-C motif) ligand 5 (CCL5; regulated on activation, normal T cell expressed and secreted (RANTES)), tumor necrosis factor (TNF)-α and NGF have been described as possible participants in the disrupted corneal homeostasis [23, 24, 26–29]. “However, it is not decided whether the alterations in the cytokine concentrations in the cornea or in tears of KC patients are the cause of the disease or an effect”[30].

To the best of our knowledge tear film components isolated from the tears of patients with PMD have not been investigated. Therefore our aim was to determine and to compare the concentrations of various mediators (IL-6, -10, CCL5/RANTES, CXCL8/IL-8, chemokine (C-X-C motif) ligand (CXCL10)/interferon-gamma-inducible protein 10 (IP-10), MMP-9, -13, TIMP-1, tissue plasminogen activator (t-PA), plasminogen activator inhibitor (PAI-1) and NGF) in the tear film of patients with PMD and KC in order to reveal any possible biochemical differences between these two entities.

## Patients and Methods

### Subjects and clinical examinations

**Fig 1:**
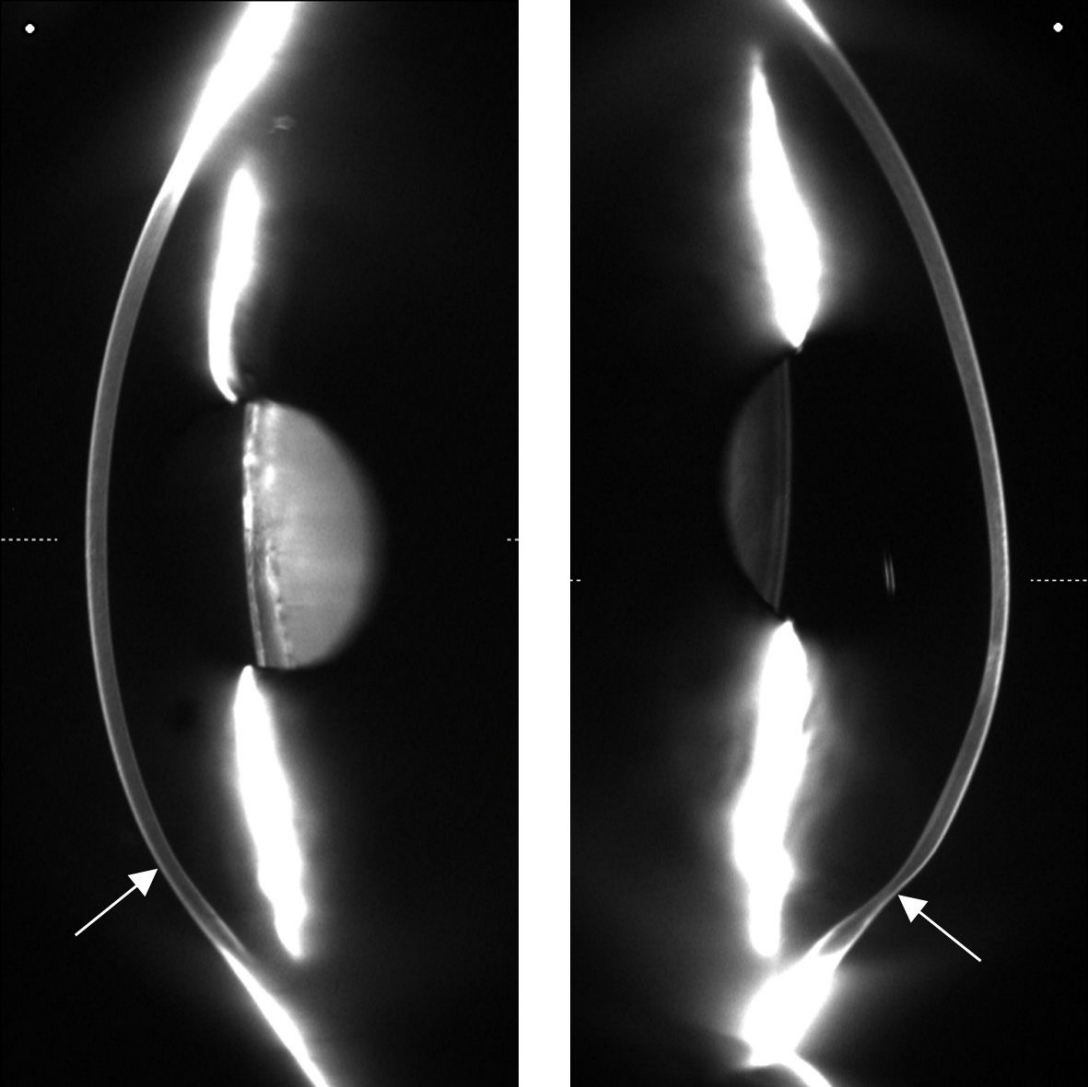
Scheimpflug images of a mild and a severe case of PMD. Arrows show the thinnest part of the cornea.

Patients with primary corneal ectasia and normal subjects were enrolled in a cross-sectional study. PMD patients with classic slit-lamp signs of PMD (an inferior, narrow band of corneal thinning separated from the limbus by a relatively uninvolved area 1–2 mm in width, and a protruding cornea above the area of thinning, associated with a flat vertical meridian and a sharp change in curvature or shape at or immediately above the band of thinning) were included. We have used the anterior and posterior elevation maps, anterior topography, Scheimpflug images (in 360-degree accurate examination mode) (Fig 1), indices and a full pachymetric map to make sure that these patients were separated from keratoconic patients, as suggested [12, 13]. We did not involve any atypical (superior) type of PMD. Non-stimulated tear samples (chemical and mechanical stimuli were not used; and care were taken to minimize ocular surface contact) were collected from nine eyes of seven PMD patients and 55 eyes of 55 KC patients. The criteria for diagnosing KC were defined as one or a combination of the following clinical signs: central or paracentral stromal thinning of the cornea, conical protrusion, Fleischer’s ring, Vogt’s striae by slit-lamp examination, as well as topographic changes [31]. We used rotating Scheimpflug tomography (Pentacam HR, Oculus Optikgeräte GmbH, Wetzlar, Germany) for the precise diagnosis of corneal ectasias. Only eyes with a 12 mm-wide measurement were included in the study with the recommended settings (that means: „limit map to 9.0 mm” setting was turned off in all cases) [32]. We do not include the fellow eye of the PMD patients without a definitive diagnosis of corneal ectasia. Because of the rarity of PMD, in the instance of this ectasia, both eyes of the patients were used if they met the aforementioned inclusion criteria. The exclusion criteria were: active inflammatory or infective systemic or ocular disease, current treatment with systemic or local drugs, use of eye drops, previous ocular surgery, abnormality in the lens or retina on biomicroscopic examination, chemical injury or delayed epithelial healing and pregnancy or lactation. Patients with allergic symptoms and patients with ocular allergic signs on slit-lamp biomicroscopy were excluded from the study. We took a detailed case history of every patient, and asymptomatic asthmatic disease and asymptomatic allergic dermatic disorders were recorded. Contact lens wearing was discontinued at least two weeks before the measurements and tear sample collection. Twenty-four randomly selected right or left eyes of 24 healthy controls were also enrolled on this study. Controls and KC patients were recruited after recruitment into the PMD group was complete. Controls were included if they were at least 29 years of age and willing to participate. The age limit was used to ensure an overlap between the groups in terms of age, without setting it so high as to render recruitment into the KC group prohibitively difficult; 29 years was judged to be a sensible cut-off. KC patients were included if they met these same two criteria, plus at least one of three additional criteria on the similarity of severity parameters. These were: the subject's K2 (Holladay equivalent keratometry value in the steep meridian) to be within the PMD group's mean K2 ± 0.2 D; Kmax (maximal keratometry of the front surface), within PMD mean Kmax ± 0.8 D; and Rmin (minimal radius of curvature), within PMD mean Rmin ± 0.1 mm. The recruitment procedure was run exhaustively on all of the potential controls and the KC patients who were available for us to contact. Following the tenets of the Declaration of Helsinki, written informed consent was signed by all the participants prior to enrolment. This study was approved by the Institutional Ethics Committee of the University of Debrecen.

All eyes received a complete ophthalmological evaluation, including corrected visual acuity measurements and slit-lamp biomicroscopy. Subsequently, three images were captured in all the study eyes with the high-resolution version of the Pentacam device (Pentacam HR, software version 1.17r139; Oculus Optikgeräte GmbH, Wetzlar, Germany) using Scheimpflug imaging. The imaging procedure was subsequently repeated in cases where any capturing error occurred (blinking, lack of data, etc.). The following data were exported to Microsoft Excel (Microsoft Corp, Redmond, Washington): Holladay equivalent keratometry values in the flat meridian (K1), K2, Kmax, corneal astigmatism, corneal thickness at the apex (Pachy Apex) and at the thinnest point of the cornea (Pachy Min), Rmin, index of surface variation (ISV), index of vertical asymmetry (IVA), keratoconus index (KI), central keratoconus index (CKI), index of height asymmetry (IHA) and index of height decentration (IHD).

### Tear collection and analysis

After the assessment of the anterior ocular status of each patient, non-traumatic tear collection was carried out in the morning between 8.00 a.m. and 9.30 a.m. with capillary tubes from the inferior meniscus, without topical anesthesia, for two minutes. The tear volume was calculated and registered accordingly [33]. All the collected tear volumes were over 4 μl. The samples were frozen at –80°C without centrifugation within 15 min following collection. A microparticle-based flow Cytometric Bead Array (CBA) technology allowing the quantification of multiple proteins in small individual tear samples was used in the study.

The concentrations of IL-6, IL-10, CCL5 (RANTES), CXCL8 (IL-8), CXCL10 (IP-10), MMP-9, MMP-13, TIMP-1, NGF, tPA and PAI-1 were measured by the CBA method. Combined FlowCytomix™ Simplex Kits were used with the appropriate FlowCytomix Basic Kit with minor modifications of the manufacturer’s instructions (eBioscience, Bender MedSystems GmbH, Vienna, Austria), as described Kolozsvári et al. [34]. We had applied additional dilutions as an extension of the recommended standard serial dilution steps. The titration curves had been computed and drawn by the help of the fluorescence intensities in the function of the dilution/concentration. The last dilution step/point (and the corresponding concentration and fluorescence intensity) before the linear part of the standard curve started to deviate into the horizontal plateau of the threshold value (corresponding also to the 0 pg/ml concentration) was used as the 'detection limit' or sensitivity of that measurement. The detection limits were, for IL-6: 1.2 pg/ml, IL-10: 1.9 pg/ml, CCL5/RANTES: 25 pg/ml, CXCL8/IL-8: 0.5 pg/ml, CXCL10/IP-10: 6.0 pg/ml, MMP-9: 95 pg/ml, MMP-13: 50 pg/ml, TIMP-1: 28 pg/ml, t-PA: 4.8 pg/ml, PAI-1: 13.5 pg/ml and NGF: 126.8 pg/ml.

### Statistical methods

**Fig 2:**
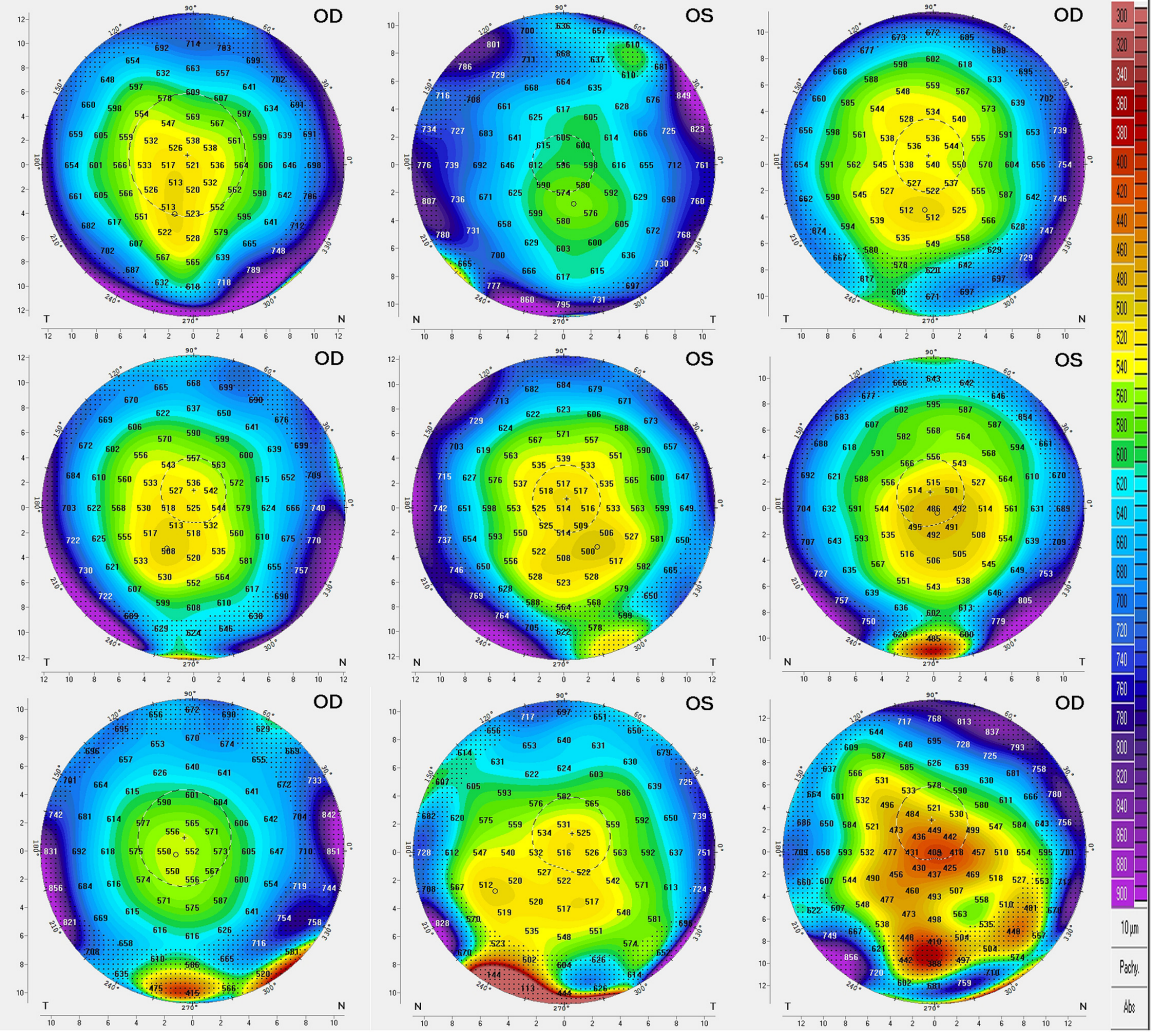
Corneal pachymetry plots of the 9 PMD eyes (from the mildest to the advanced cases).

**Fig 3:**
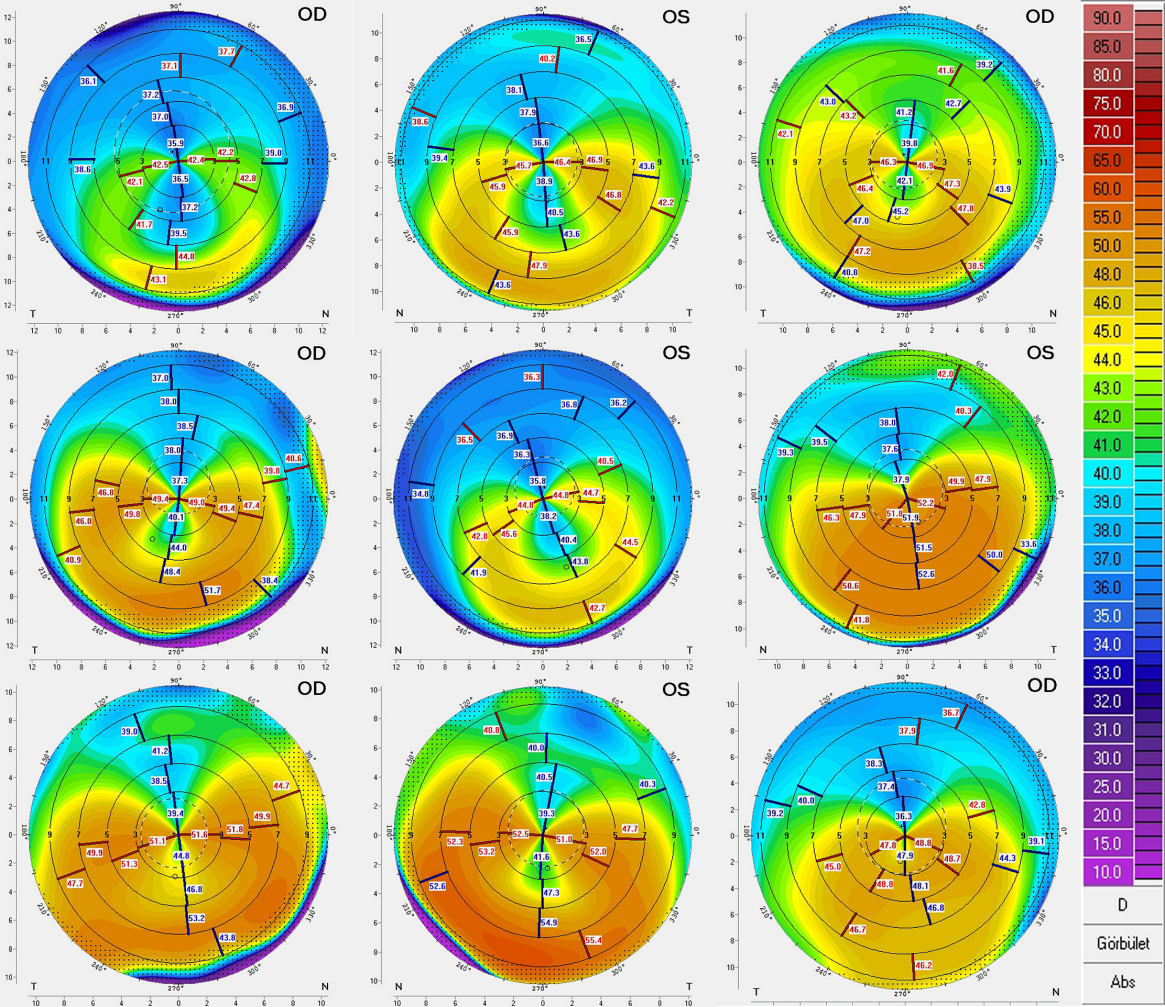
Corneal topographical maps of the 9 PMD eyes (in similar order than in Fig 2).

Only one eye per patient in the keratoconus and normal groups was analyzed to avoid bias due to inter-eye correlations. Conversely, both eyes of the PMD patients were analyzed (Figs 2 and 3) due to the rarity of this corneal ectasia. Statistical methods appropriate for the presence of eyes nested within subjects were used. The variables were described in terms of means and SD on their native scales.

**Fig 4:**
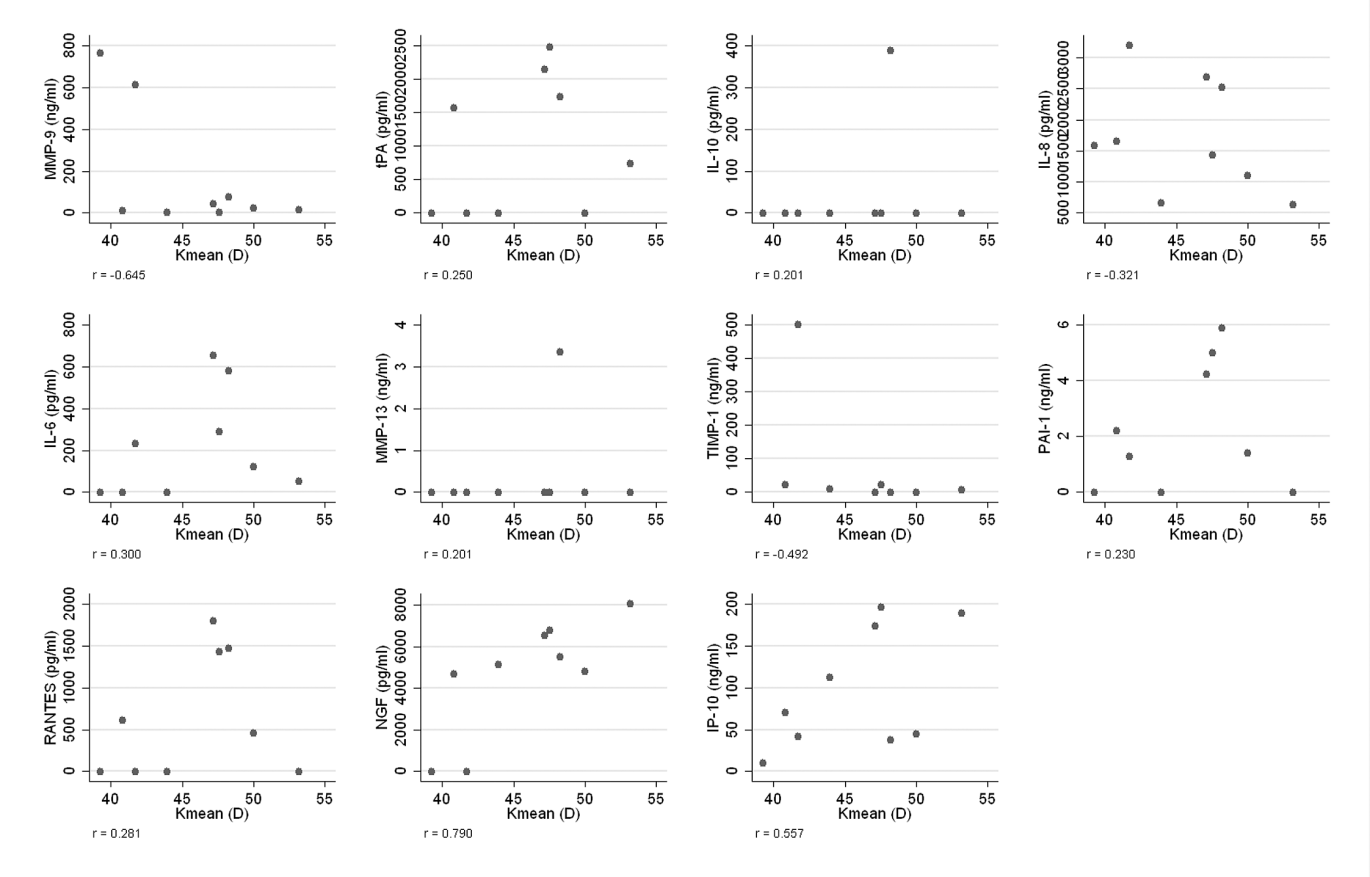
Scatter plots of mediator concentrations against the average of the parameters K1 and K2 (r: Pearson's correlation coefficient).

PMD patients' tear film mediator concentrations were plotted against mean K values, and Pearson's correlation coefficients were calculated, to evaluate the relationship between such pairs of variables (Fig 4).

Multilevel mixed-effects linear regression was used to compare the patient groups in terms of mediator concentrations and keratometric readings obtained from a Scheimpflug camera (K1, K2, Kmax (front), Astigmatism, Pachy Apex, Pachy Min, Rmin, KI, CKI, IHA, ISV, IVA, IHD). Models were fitted separately for each outcome. The statistical package applied was Stata version 11. The significance criterion was set at α = 0.05.

## Results

The values of the parameters measured with Pentacam in the patient groups and the control group (PMD group: mean age: 46.4 years, range 36–59 years; KC group: mean age: 44.2 years, range 29–61 years; control group: mean age: 44.5 years, range: 29–67 years) and the differences between the groups are shown in Tables 1 and 2. We enrolled the eyes of keratoconic patients who were similar to the PMD patients not only in age but also in severity (K2, Kmax and Rmin), which explains the absence of significant differences not only in K2, Kmax and Rmin but also in Pachy Apex, Pachy Min, KI, IHA, ISV and IVA.

**Table 1:** Mean±1SD values of the parameters measured with Pentacam in patients with pellucid marginal degeneration, keratoconus and in healthy controls.

	PMD (9 eyes of 7 patients)	KC (55 eyes of 55 patients)	Control (24 eyes of 24 participants)
**K1 F (D)**	42.4±5.6	45.6±4.0	43.4±1.4
**K2 F (D)**	49.1±4.0	49.3±4.1	44.3±1.6
**Kmax**	55.2±7.2	54.4±5.4	44.8±1.6
**Astigmatism (D)**	6.7±2.9	3.7±1.9	0.9±0.6
**Rmin (mm)**	6.2±0.8	6.3±0.6	7.6±0.3
**Pachy Apex (μm)**	502±47	486±49	551±32
**Pachy Min (μm)**	489±45	468±51	545±33
**ISV**	97.9±45.6	80.8±31.3	15.1±4.4
**IVA**	1.04±0.51	0.86±0.42	0.11±0.04
**KI**	1.24±0.23	1.21±0.12	1.02±0.02
**CKI**	1.01±0.34	1.04±0.04	1.00±0.01
**IHA**	17.8±11.5	23.3±19.7	3.8±2.4
**IHD**	0.12±0.08	0.08±0.05	0.01±0.00

Patient groups: PMD = pellucid marginal degeneration; KC = keratoconus. Pentacam parameters: Holladay equivalent keratometry values in the flat (K1) and steep (K2) meridian, maximal keratometry of the front surface (Kmax), minimal radius of curvature (Rmin), corneal thickness at the apex (Pachy Apex), at the thinnest point of the cornea (Pachy Min), index of surface variation (ISV), index of vertical asymmetry (IVA), keratoconus index (KI), central keratoconus Index (CKI), index of height asymmetry (IHA), index of height decentration (IHD).

**Table 2:** Differences between the parameters measured with Pentacam among the patient groups.

	K1	Astigmatism	K2, Kmax, Rmin, Pachy Apex, Pachy Min, ISV, IVA, KI	CKI	IHD	IHA
**PMD v. Control**	p = 0.312	**p<0.0001**	**p<0.0048**	p = 0.834	**p<0.0001**	**p = 0.0465**
**KC v. Control**	**p = 0.01**	**p<0.0001**	**p<0.0001**	**p<0.0001**	**p<0.0001**	**p<0.0001**
**PMD v. KC**	**p = 0.006**	**p<0.0001**	p≥0.0814	**p = 0.004**	**p = 0.038**	p = 0.224

Patient groups: PMD = pellucid marginal degeneration; KC = keratoconus. Pentacam parameters: Holladay equivalent keratometry values in the flat (K1) and steep (K2) meridian, maximal keratometry of the front surface (Kmax), minimal radius of curvature (Rmin), corneal thickness at the apex (Pachy Apex), at the thinnest point of the cornea (Pachy Min), index of surface variation (ISV), index of vertical asymmetry (IVA), keratoconus index (KI), central keratoconus Index (CKI), index of height asymmetry (IHA), index of height decentration (IHD) (bold values represent significant associations).

The concentrations of the mediators in the tear film of patients in the different patient groups and the differences between the groups are shown in Tables 3 and 4. MMP-9 was the only mediator that significantly differed between the two ectatic patient groups (p = 0.005). There was no evidence of a relationship between mediator concentrations and mean K values (Fig 2).

**Table 3:** Mean±1SD concentrations of the mediators in the tear film of patients.

	PMD (9 eyes of 7 patients)	KC (55 eyes of 55 patients)	Control (24 eyes of 24 participants)
**IL-6 (pg/ml)**	213±251.2	160.2±265.7	217.1±172.2
**IL-10 (pg/ml)**	43.06±129.2	280.3±832.8	625.1±708.2
**CCL5/RANTES (pg/ml)**	636.5±731.2	412.5±542.1	218.8±194.1
**CXCL8/IL-8 (pg/ml)**	1719±905.8	2231±2857	4026±2681
**CXCL10/IP-10**	96.9±72.4	91.9±87.3	124.7±123.3
**MMP-9 (ng/ml)**	170.8±294.7	51.3±131.9	36.7±61.5
**MMP-13 (ng/ml)**	0.371±1.1	36.6±88.0	96.7±77.1
**TIMP-1 (ng/ml)**	69.7±174	127.1±251.7	160.9±164.6
**tPA (pg/ml)**	960±1023	4066±8545	7304±5737
**PAI-1 (ng/ml)**	2.21±2.3	2.08±2.6	2.16±2.1
**NGF (pg/ml)**	4603±2823	4523±4266	3160±2584

Patient groups: PMD = pellucid marginal degeneration; KC = keratoconus. Mediators: interleukin (IL)-6, IL-10, chemokine (C-C motif) ligand 5 (CCL5)/regulated and normal T cell expressed and secreted (RANTES), chemokine (C-X-C motif) ligand (CXCL)8/IL-8, CXCL10/ interferon-gamma-inducible protein 10 (IP-10), matrix metalloproteinase (MMP)-9, MMP-13, tissue inhibitor of metalloproteinases-1 (TIMP-1), tissue plasminogen activator (t-PA), plasminogen activator inhibitor (PAI-1) and nerve growth factor (NGF).

**Table 4:** Differences between the concentrations of the mediators in the tear film of patients among the patient groups.

	IL-8	MMP-9	MMP-13	t-PA
**PMD v. Control**	**p = 0.031**	**p = 0.004**	**p = 0.004**	**p = 0.031**
**KC v. Control**	**p = 0.011**	p = 0.655	**p = 0.004**	p = 0.094
**PMD v. KC**	p = 0.479	**p = 0.005**	p = 0.198	p = 0.226

Patient groups: PMD = pellucid marginal degeneration; KC = keratoconus. Mediators: interleukin (IL)-8/chemokine (C-X-C motif) ligand (CXCL)8, matrix metalloproteinase (MMP)-9, MMP-13, tissue plasminogen activator (t-PA) (bold values represent significant associations).

Although we could not establish relevant differences because of the high standard deviations, the anti-inflammatory cytokine IL-10 was 14-times lower in PMD than in the controls, and seven-times lower than in KC. In addition, CCL5, which is chemotactic for T cells, eosinophils and basophils, and which plays an active role in recruiting leukocytes into inflammatory sites, was the highest in PMD compared to the two other groups. In line with this, TIMP-1, one of the most important MMP-inhibitors, was the lowest in PMD as compared to KC and the controls. The ratios of MMP-9 and TIMP-1 were 2.45, 0.40 and 0.23 in PMD, KC and the controls, respectively. In contrast with these findings, MMP-13 and tPA were the lowest in the PMD group.

## Discussion

To the best of our knowledge, this is the first study that has aimed to reveal the biochemical differences between PMD and KC. Most of the ectatic corneal disorder cases are KC, and although PMD is far rarer it is no less important [11]. PMD and KC differ relevantly in terms of prognosis and management, and distinguishing between these two conditions is of potential clinical importance [8, 13, 15, 35]. If biomarkers in the tear film can help in differentiating between the two entities, this non-invasive procedure could be advocated in the diagnostic tree. There are reports suggesting that PMD, KC and keratoglobus may belong on a spectrum of the same pathophysiology rather than separate disease processes [36, 37], but based on our preliminary results it seems that PMD and KC are distinct diseases and might not be the phenotypic variations of the same disorder.

In moderate cases, PMD can be distinguished from KC by slit-lamp biomicroscopic examination due to the classic location of the region of corneal thinning [7]. Although elevation-based Scheimpflug imaging shows the hallmarks of classic PMD, and an accurate diagnosis could be made with images of the peripheral cornea and PMD could be clearly identified and separated from KC, there are no specific or universally accepted topographic criteria for categorizing an eye as having PMD [35].

In this study we evaluated the concentration of different mediators in tear samples. MMPs are secreted in response to cytokines and growth factors, and elevated levels of MMP-9 in the tear fluid of PMD and KC patients indicate a tissue-degenerative process contributing to the thinning of the cornea. MMP-9 was the only mediator which significantly differed between PMD and KC (p = 0.005). Our preliminary examination (data not presented) has shown that the concentration of MMP-9 in the tears of keratoconic patients did not alter significantly with age. The more elevated MMP-9 in the tear fluid of PMD patients compared to KC patients might suggest a basic difference in the underlying pathological tissue-degradative processes. Elevated levels of MMP-1, -3, -7, -9 and -13 have been found in the tears of patients with KC [24, 27, 28]. The ocular surface inflammation is activated by proteases, including MMP-9 [38, 39], and based on our study it may help to differentiate between keratoconus and pellucid marginal degeneration. MMPs and cytokines interact with each other by forming a complex network, including the stimulation of MMP-9 and MMP-13 by IL-6 [27]. Strongly expressed MMP-13 was reported in KC suggesting a role in intra- and extracellular pathological collagen destruction [40]. In contrast with this finding, we found a significantly lower MMP-13 concentration (p = 0.004) in the tears of patients with both disorders. Although the difference was not statistically significant, patients with PMD had the lowest MMP-13 concentration, which should be further investigated because of the complexity of the cascade of the degenerative processes. The active form of t-PA converts plasminogen to plasmin, which can also degrade several components of the extracellular matrix and trigger the activation of the MMP pathway. MMPs and PAs in turn are partially regulated by TIMPs and PAIs, inhibiting this cascade system and therefore influencing the progression of ectatic corneal diseases. In earlier studies, non-specific, slightly elevated t-PAs were detected in KC, reflecting the different corneal healing processes, but until now there have been no studies examining this protein in PMD [34, 41]. The PAI-1 gene can be induced by several growth factors and cytokines, and it can inhibit the activity of t-PA enzymes. Interestingly, in our study, the t-PA concentration was significantly lower in PMD compared to the controls while the PAI-1 concentrations were the same in the three groups, which suggests that other enzymes might play a more important role in the underlying molecular mechanism, and probably that the actual enzyme activities influence the final-effect impact on tissue degradation in ectatic corneal disorders. TIMPs are natural inhibitors of the different MMPs, and there have been various studies presenting conflicting reports on the expression of TIMP-1 in keratoconic corneas [25, 27, 42, 43]. In our study, TIMP-1 (one of the most important MMP inhibitors) was the lowest in PMD as compared to KC and the controls, and this reduced activity might have an impact on tissue degradation. Evaluating the balance between MMP-9 and TIMP-1, the ratios of MMP-9 and TIMP-1 were 2.45, 0.40 and 0.23 in PMD, KC and the control groups, respectively, indicating a more pronounced tissue degradation in PMD than in KC. Future studies may be needed to confirm the active interplay between MMPs and TIMPs as well as ILs, especially IL-10.

KC is defined as a non-inflammatory disease of the cornea, but an increasing number of studies have shown the role of the over-expression of several cytokines [26, 27]; therefore, classifying the disease as ‘non-inflammatory’ may now be inappropriate [20, 25–27, 44]. In contrast with these previous findings based on younger keratoconic patients, in our study IL-6 in the tears of ectatic patients and in the controls did not differ significantly [24, 26–28, 44]. Additionally, elevated levels were detected in inflammatory ocular surface diseases as well as in keratoconus [27, 45], and CXCL8, which is the predominant chemoattractant in the tear fluid, was significantly lower both in PMD and KC than in the controls in our study. We could detect that CCL5, which is chemotactic for T cells, eosinophils and basophils, and which plays an active role in recruiting leukocytes into inflammatory sites, was highest in PMD as compared to the two other groups. Our finding that in both corneal ectasias the concentrations of CCL5 are higher than normal are in contrast with Jun et al. [26]. Additional studies are needed to further validate the role of IL-6, CXCL8 and CCL5 in the pathomechanism of corneal ectasias and in tissue damage in PMD and KC. The anti-inflammatory cytokine, IL-10, was 14-times lower in PMD than in the controls and seven-times lower than in KC. The lower concentration of the anti-inflammatory cytokine IL-10 in the tear fluid of PMD patients compared with keratoconic patients and with the controls in the current study supports the assumption that cytokines and chemokines play an important role in the pathomechanism of KC. IP-10/CXCL10 is a fibrotic and angiostatic chemokine produced by macrophages, endothelial cells and fibroblasts [46], and it did not show significant differences between our patient groups. Our observation supports the fact that the cornea in KC is avascular and that scarrring occurs only in the severe stage, which was presented in our cohort in just 11%. It has been suggested that the impairment of corneal innervation has a role in the pathogenesis of KC, and a significant reduction in NGF expression has been detected in KC corneas as part of an imbalance in the NGF signaling pathway [47, 48]. Our results are in contrast with the previous findings because we could not establish differences in the concentration of NGF between the patient groups; nor were we able to find any correlation between the severity of KC and NGF [49].

The limitations of our study are the small number of patients with PMD and the fact that only older patients (mean ages over 44 years) were included. However, because of the rarity of pellucid marginal degeneration, the nine eyes with PMD involved in this study are a proportional representation of our around four hundred ectatic patient population. The cause of the older patient age is that the manifestations of PMD are evident at a later age, and we wanted to involve true PMD cases in this study. From this point of view, we could have compared PMD patients with KC cases of an older age, when keratoconus is less progressive. It also would have been desirable to involve the early stages of PMD among younger people in the investigation, because of the possible progression of the disease which can be accompanied with alterations in the mediator levels. Furthermore, a limitation of this study is that it cannot exclude the possibility of other mediators being involved in the pathomechanism of PMD nor the identification of the source and activity of the mediators (including MMP-9) and the expression of the different receptors. Although it has been demonstrated that mediators reveal the biochemical differences between pellucid marginal degeneration and keratoconus, it is unclear which proteins can be used to distinguish the non-progressive form of corneal ectasia from the progressive type (where early treatment would be very important).

Tear samples are an essential tool to understand the molecular mechanism behind corneal ectasias, and the multiplex platform is ideally suited for the detection of biomarkers from the small volumes of tear samples. It remains to be determined in further studies which of these mediators are critical in differentiating between these two entities.

Our results show for the first time the significant differences in the levels of MMP-9 in tear samples between these two ectatic corneal disorders. Although the morphological difference between the two diseases has already been reported by several previous studies [4, 5, 11, 15], this is the first high-throughput research demonstrating the differences in the levels of several proteins, including cytokines and enzymes (with inhibitors), in tear samples.

Further studies with a larger sample might be required to validate these preliminary findings and to demonstrate the importance of MMP-9. Cytokines, chemokines and enzymes are important in regulating wound healing, apoptosis, cell cycling and migration processes under physiological or pathological conditions. It is assumed that the biomarkers from this complex network and examined in our study and others may be responsible for disease progression.

In summary, this study reveals several mediators being altered in the tears of PMD and KC patients, and these alterations may have an impact on the differences between these ectatic diseases. Additionally, research is required to further elucidate the differences and the importance of mediators in the pathomechanism of PMD and KC.
